# Multicolour three dimensional structured illumination microscopy of immunolabeled plant microtubules and associated proteins

**DOI:** 10.1186/s13007-019-0406-z

**Published:** 2019-03-09

**Authors:** T. Vavrdová, O. Šamajová, P. Křenek, M. Ovečka, P. Floková, R. Šnaurová, J. Šamaj, G. Komis

**Affiliations:** Department of Cell Biology, Centre of the Region Haná for Biotechnological and Agricultural Research, Faculty of Science, Palacký University Olomouc, Olomouc, Czech Republic

**Keywords:** Immunofluorescence, Microtubules, Microtubule associated proteins, Structured illumination microscopy

## Abstract

**Background:**

In the present work, we provide an account of structured illumination microscopy (SIM) imaging of fixed and immunolabeled plant probes. We take advantage of SIM, to superresolve intracellular structures at a considerable z-range and circumvent its low temporal resolution capacity during the study of living samples. Further, we validate the protocol for the imaging of fixed transgenic material expressing fluorescent protein-based markers of different subcellular structures.

**Results:**

Focus is given on 3D imaging of bulky subcellular structures, such as mitotic and cytokinetic microtubule arrays as well as on the performance of SIM using multichannel imaging and the quantitative correlations that can be deduced. As a proof of concept, we provide a superresolution output on the organization of cortical microtubules in wild-type and mutant Arabidopsis cells, including aberrant preprophase microtubule bands and phragmoplasts in a cytoskeletal mutant devoid of the p60 subunit of the microtubule severing protein KATANIN and refined details of cytoskeletal aberrations in the mitogen activated protein kinase (MAPK) mutant *mpk4*. We further demonstrate, in a qualitative and quantitative manner, colocalizations between MPK6 and unknown dually phosphorylated and activated MAPK species and we follow the localization of the microtubule associated protein 65-3 (MAP65-3) in telophase and cytokinetic microtubular arrays.

**Conclusions:**

3D SIM is a powerful, versatile and adaptable microscopy method for elucidating spatial relationships between subcellular compartments. Improved methods of sample preparation aiming to the compensation of refractive index mismatches, allow the use of 3D SIM in the documentation of complex plant cell structures, such as microtubule arrays and the elucidation of their interactions with microtubule associated proteins.

**Electronic supplementary material:**

The online version of this article (10.1186/s13007-019-0406-z) contains supplementary material, which is available to authorized users.

## Background

Fluorescence microscopy, is the most powerful method to specifically interrogate subcellular infrastructure and the dynamics of distinct compartments and macromolecular assemblies [9]. Depending on the various confocal or widefield platforms that exist and the labeling strategies employed, it is possible to address intracellular architecture and dynamics of specific compartments at a wide range of spatiotemporal resolution (e.g., [24, 36]).

The need of imaging subcellular architecture, necessitates microscopy approaches with adequate resolving power at all three dimensions, since many such structures occupy a significant volume. 3D imaging has to cope with issues of light scattering due to refractive index mismatches and out-of-focus signal (e.g., [56]). Confocal laser scanning microscopy (CLSM), is being routinely used for 3D imaging, as it provides optical sectioning and exclusion of out-of-focus light by means of an adjustable pinhole [19]. However, CLSM in practice has very poor resolving capacity that may be slightly below 300 nm at the XY-plane and more than twice as much at the Z-dimension depending on the excitation light wavelength, the numerical aperture of the collecting objective and the pinhole settings (e.g., [8, 19, 24] and references therein), while for volumetric imaging, CLSM is time limited, although this issue is currently circumvented with resonant scanning at the expense of spatial resolution [19].

The diffraction limitations of widefield or confocal scanning fluorescence microscopy methods have been overcome to a considerable extend by different superresolution modalities [23, 24]. Such methods, convey structural information below Abbe’s diffraction limits, either by patterned light illumination strategies (such as structured illumination microscopy, SIM; [15], or stimulated emission depletion microscopy, STED; [16]), while others exemplified by photoactivation localization microscopy (PALM; [7]) and the closely related stochastic optical reconstruction microscopy (STORM; [41]), utilize photochemical properties of fluorophores and highly sensitive acquisition systems to improve the precision of localization of individual molecules. The dimensional resolution using such methods can be isotropic (as is the case with STED and PALM/STORM), or slightly inferior at the Z-dimension (as in the case of SIM; [23]).

Within limits, all superresolution methods are able to resolve subcellular structures in living cells labeled with genetically encoded fluorescent markers but they differ in both terms of temporal resolution and their effective z-range.

Unlike TIRF-based methods such as PALM or dSTORM, SIM is a widefield approach that can offer superresolution at large fields of view (FOVs). This allows imaging of considerable tissue areas and facilitates quantitative deductions of the subcellular distribution of organelles in many cells. Moreover, employment of 3D-SIM via commercially available SIM platforms can offer an effective z-range of more than 10 μm (e.g., [55]), allowing the documentation of voluminous subcellular structures such as interphase nuclei [13, 26, 47, 49–51], mitotic chromosomes [1, 3, 29, 39, 48], the meiotic and mitotic spindle [29, 57], or plasmodesmata organization [14] with a z-resolution which can be easily half that of CLSM (reviewed in [42]). In all commercially available SIM platforms, volumetric imaging is limited by the temporal component, suggesting that imaging of structures occupying considerable volumes, is best done in stabilized and appropriately labeled cells. Until recently, commercial SIM platforms (including Zeiss Elyra S.1, Nikon NSIM and Deltavision OMX), presented limitations in the extend of volumetric imaging, either with restrictions of the z-range (which for all platforms is between 5 and 15 µm), acquisition speed (which can be very limiting for volumetric imaging), or channel range which can be visualized (e.g., the configuration of Elyra S.1 allows only one camera by default meaning that multichannel imaging requires time consuming sequential acquisition). Such problems have been largely resolved with the new generation SIM microscopes such as the Zeiss Elyra 7 and the Deltavision OMX SR. By using unique modes of illumination (e.g., with lattice illumination in Elyra 7 and interferometric light pattern generation in the Deltavision OMX SR) and default configurations which may include from 2 up to 4 cameras, they allow very high acquisition frame rates (between 255 fps for Elyra 7 and 400 fps for OMX SR) and simultaneous multichannel imaging, suitable for high-throughput volumetric imaging of multiple labeled samples at affordable time scales (https://www.zeiss.com/microscopy/int/products/elyra-7-with-lattice-sim-for-fast-and-gentle-3d-superresolution-microscopy.html; https://www.gelifesciences.com/en/us/shop/cell-imaging-and-analysis/high-and-super-resolution-microscopes/instruments/deltavision-omx-sr-imaging-system-p-03020). It should be noted, however, that SIM relies on computer-assisted image reconstruction which is prone to several errors that might arise from the quality of the sample and principally from insufficient signal-to-noise ratios (e.g., due to poor labeling, high background etc.). Most notably, 3D-SIM is prone to artefactual reconstruction owing to spherical aberrations [2, 12, 25] from refractive index mismatches in the optical path.

We have routinely used and established protocols for live cell imaging of plant cells expressing fluorescent protein-based markers of different subcellular structures in order to extrapolate and quantify dynamic features of microtubules, actin filaments and endoplasmic reticulum among others [22, 24, 25]. Imaging of fixed and immunolabeled samples offers the advantage of signal amplification compared to fluorescent proteins and allows the elucidation of details in subcellular architecture that would have escaped the capacity of live cell imaging. We show case studies as proofs-of-principle on the efficiency of the preparatory labeling protocol, and potential biological outputs of 2-D, 3-D and multichannel SIM imaging. In particular, we show how 2D SIM can be used to resolve and quantify the organization of widespread intracellular structures such as cortical microtubules, the potential of 3D SIM to document subcellular structures with considerable three dimensional occupancy, and some applications of multichannel imaging with emphasis on the use of multicolor SIM in colocalization experiments.

## Results

### Validation of the immunolabeling procedure

By carefully controlling fixation and enzymatic digestion of the cell walls, it was possible to maintain the cellular and tissue integrity of the root tip, to structurally preserve microtubular appearance and integrity and to allow primary and secondary antibodies to diffuse throughout the entire volume of the sample. By increasing the stringency of blocking by prolonging blocking time and using a combination of blocking agents (BSA and BSAc) it was possible to reduce background fluorescence and to increase signal-to-noise ratio (SNR) to levels adequate for high quality SIM imaging which is particularly important for diffusely distributed antigens such as MAPK species. Finally, the use of controlled RI mounting media adequately compensated for spherical aberrations that may hamper SIM image acquisition at considerable RI mismatches during 3D acquisition.

### Cortical microtubule organization in Col-0, *mpk4* and *ktn1*-*2*

Microtubule appearance, integrity and overall organization is a classical example to demonstrate labeling efficiency of a labeling and imaging process. For this reason we followed and documented microtubular structures in root whole-mount preparations by means of SIM. With optimized conditions of sample preparation, mounting and imaging, we acquired a series of images corresponding to microtubule organization of interphase root cells in either wild-type Col-0 root tips, or in cells of *ktn1*-*2* and *mpk4* mutants, in order to identify minute details that may underlie the phenotypes of the respective mutants and quantify microtubule angular distribution [5, 6, 21].

**Fig. 1:**
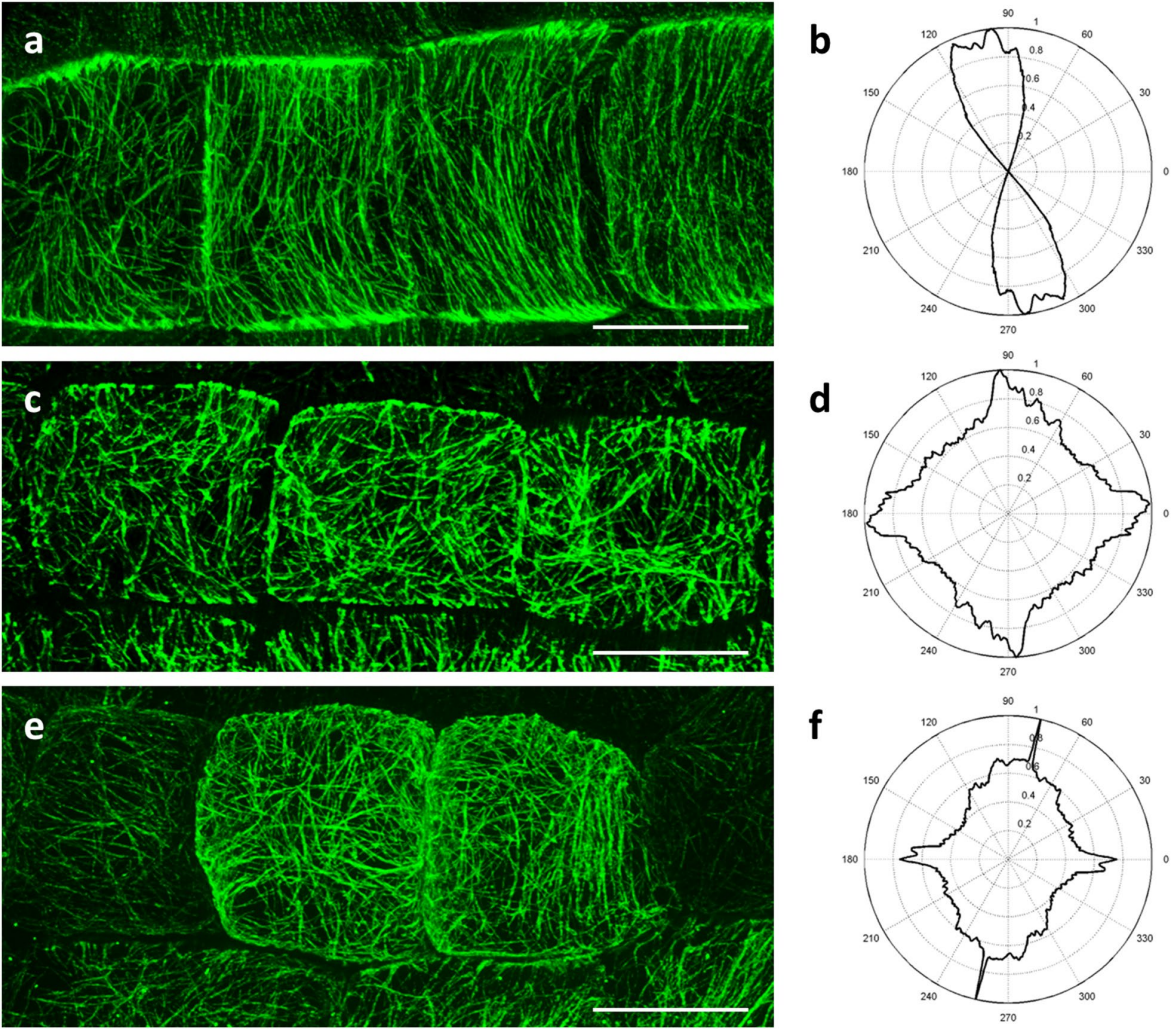
SIM imaging of cortical microtubules in Col-0, *ktn1*-*2* and *mpk4*. **a**, **b** Cortical microtubules of Col-0 meristematic root epidermal cells are well organized in parallel cortical arrays (**a**) and are predominantly transverse to slightly oblique with respect to the cell axis (**b**). In meristematic root epidermal cells of *ktn1*-*2* mutant cortical microtubules exhibit random orientations (**c**) which is reflected to their broad angular distribution (**d**). Similarly, cortical microtubules of *mpk4* root epidermal cells show mixed orientations (**e**) and broad angular distribution (**f**). Bars in **a**, **c**, **e** = 10 μm

In interphase cells cortical microtubules are predominantly parallel to each other (Fig. 1a), showing a quite narrow angular distribution at angles perpendicular or slightly oblique with respect to the cell axis (Fig. 1b). In such cells of the *ktn1*-*2* mutant, cortical microtubule systems are disorganized without a prevalent pattern (Fig. 1c) and this is also reflected by their broader angular distribution (Fig. 1d). Similarly, cortical microtubules of *mpk4* root epidermal cells (Fig. 1e) are disorganized without prevalent orientation which is again evident from their broad angular distribution (Fig. 1f).

### PPB organization in Col-0 and *ktn1*-*2*

**Fig. 2:**
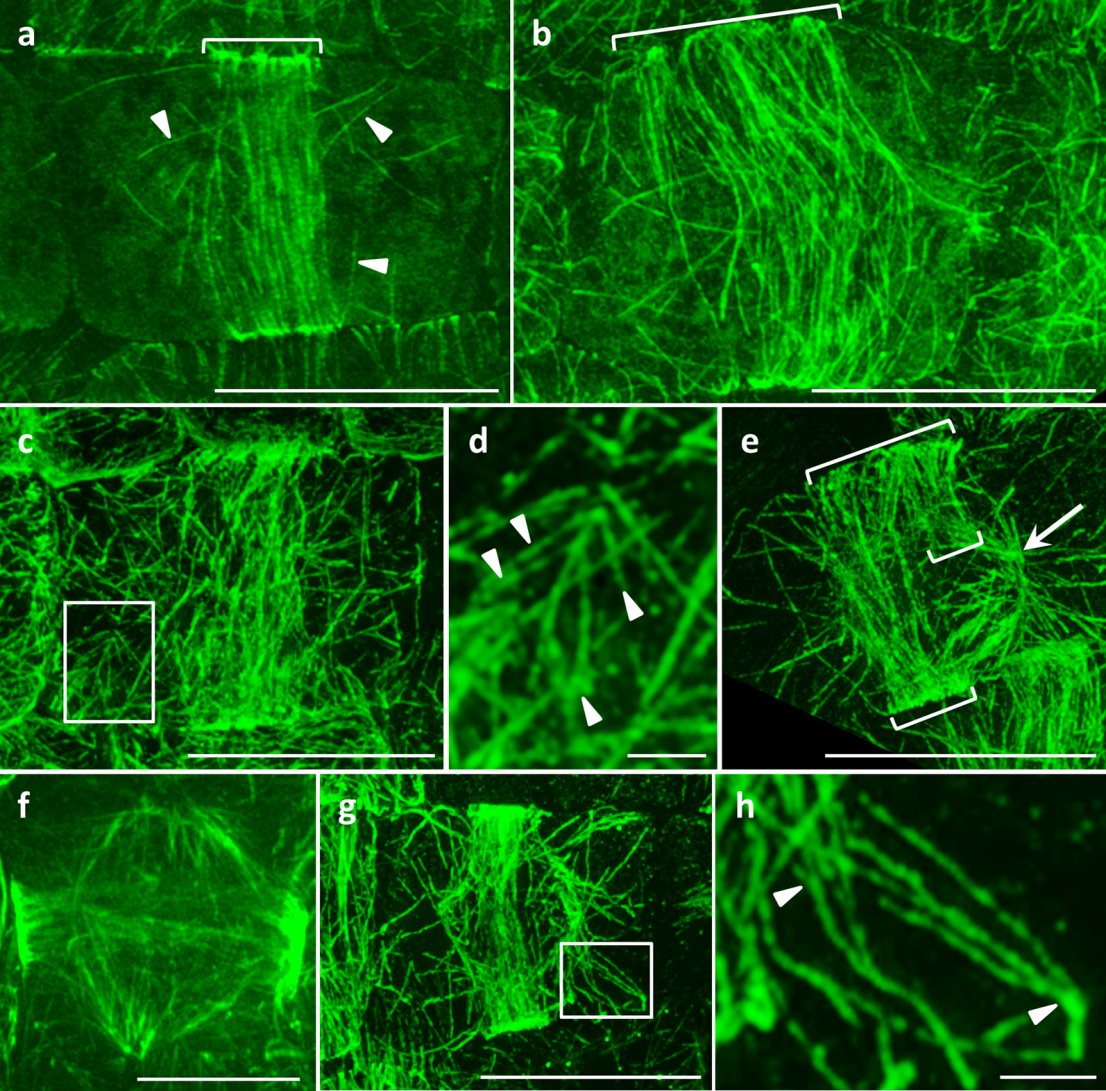
SIM imaging of PPB formation in Col-0 and *ktn1*-*2*. **a** Early PPB in Col-0 (bracket). With few exceptions (arrowheads) the cortical cytoplasm outside of the PPB is cleared from microtubules. **b** An example of a very broad PPB (bracket) in *ktn1*-*2*. **c**, **d** Another example of an early *ktn1*-*2* PPB showing a wealth of cortical microtubules outside the PPB (**c**, rectangle) and excessive microtubule branching (**d**; arrowheads) in some instances. **e** An abnormally broad late PPB (long bracket) of an early prophase *ktn1*-*2* cell showing an incomplete part (short brackets) and the formation of perinuclear spindle (arrow). **f** By comparison to (**e**), a late PPB of a Col-0 early prophase cell is shown. PPB is considerably more narrow. **g**, **h** Overview (**g**) and detail (**h**) of another abnormal preprophase cell of *ktn1*-*2* showing excessive de novo branched formation of microtubules (arrowheads). Bars in **a**–**c**, **e**, **g** = 10 μm; **f** = 5 μm; **d**, **h** = 1 μm

In a previous study we followed the dynamic course of PPB formation and organization in *ktn1*-*2* mutants which exhibit an aberrant cell division plane orientation. In that study we identified that PPB shows marked delays in its progressive narrowing and that this is accompanied by reduced clearing of cortical microtubules outside the PPB region [21]. Herein, we addressed PPB organization in fixed and immunolabeled preprophase root cells of Col-0 (Fig. 2a) and *ktn1*-*2* mutant (Fig. 2b–e, g, h). Consistent with absent severing activity owing to the lack of the p60 subunit of KATANIN, PPBs in *ktn1*-*2* mutant appear considerably broader than those of Col-0 (Fig. 2b, c.f. Fig. 2a). In particular the PPB width of Col-0 preprophase cells was 3 ± 0.9 µm (mean ± SD; N = 50) while in *ktn1*-*2* mutants it was nearly twice as much (5.5 ± 2 µm; mean ± SD; N = 22). In all cases examined, the cortical cytoplasm surrounding the PPB area is populated with microtubules unlike what is observed in wild-type cells (Fig. 2b, c, e, g, c.f. Fig. 2a). Closer inspection of such cells showed that among others, the occurrence of such cortical microtubules is owing to excessive microtubule branching formation (Fig. 2c, d, g, h). Even at later stages when a clear bipolar spindle forms around the nucleus and the PPB is considerably narrower in Col-0 (Fig. 2b, c.f. Fig. 2a), the PPB of *ktn1*-*2* remains broad as in earlier stages of its formation (Fig. 2e, g, c.f. Fig. 2f).

### Volumetric imaging of the mitotic spindle and the phragmoplast

Imaging of the mitotic spindle and the cytokinetic phragmoplast requires a considerable z-range capacity of the microscope since the spindle may expand at a depth of ca. 5 µm while the fully expanded phragmoplast may exceed 15–20 µm when it is occupying the entire circumference of the cell. As discussed earlier, 3D-SIM is very much prone to reconstruction artefacts due to spherical aberrations [2, 12]. For the purpose of mitotic spindle and phragmoplast visualization by means of 3D-SIM, we mounted immunolabeled whole-mount preparations in a thiodiethanol-based mounting medium supplemented with paraphenylene diamine as an antifade agent, at a TDE concentration that approximates the refractive index of the glass and the immersion oil (i.e., ca. 97% v/v with RI ≈ 1.52; [54]). Other studies have reported that Vectashield mounting medium may be used for 3D SIM acquisitions at a z-range of approximately 10 µm ([51] and references therein; [4] and references therein).

**Fig. 3:**
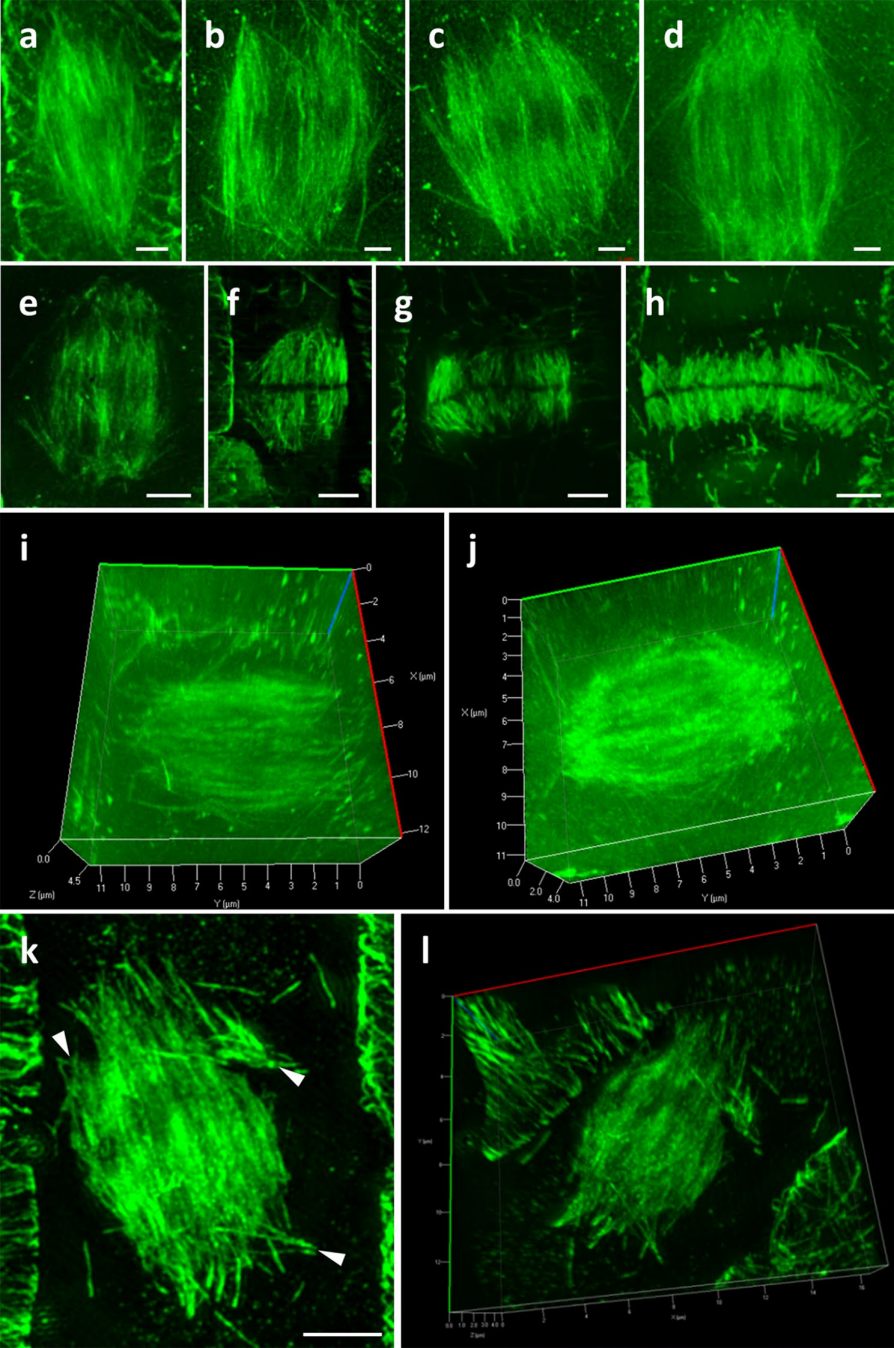
Potential of 3D-SIM in visualizing mitotic and cytokinetic microtubule configurations. **a** Maximum intensity projection of a prophase spindle. **b** Maximum intensity projection of a prometaphase spindle. **c** Maximum intensity projection of a metaphase spindle. **d** Maximum intensity projection of an early anaphase spindle. **e** Maximum intensity projection of a telophase spindle. **f**–**h** Successive stages of centrifugal phragmoplast expansion. **i**, **j** Examples of 3D rendering of cells depicted in **b** and **d** respectively (see also Additional file 1: Video 1 and Additional file 2: Video 2), showing the potential of SIM to capture the entire volume of the mitotic apparatus. **k**, **l** Maximum intensity projection (**k**) and 3D (**l**) rendering of an aberrant mitotic spindle of *ktn1*-*2* (see also Additional file 3: Video 3). Arrowheads show ectopic microtubules occurrence (**k**). Bars in **a**–**h**, **k**, **l** = 2 μm

In such immunolabeled root whole-mount preparations of Col-0 we were able to document all the stages of the mitotic spindle including prophase (Fig. 3a), prometaphase (Fig. 3b), metaphase (Fig. 3c), anaphase (Fig. 3d) and telophase (Fig. 3e) in the three dimensions. During cytokinesis, it was possible to follow all stages of phragmoplast formation (Fig. 3e–h). Owing to refractive index compensations it was possible to follow the entire volume of the mitotic spindle as exemplified by appropriate 3D rendering (e.g., Figure 3i, j; Additional file 1: Video 1 and Additional file 2: Video 2). Finally 3D-SIM was used to document an aberrant mitotic spindle of *ktn1*-*2* mutant with ectopic microtubule occurrence (Fig. 3k, l; Additional file 3: Video 3).

**Fig. 4:**
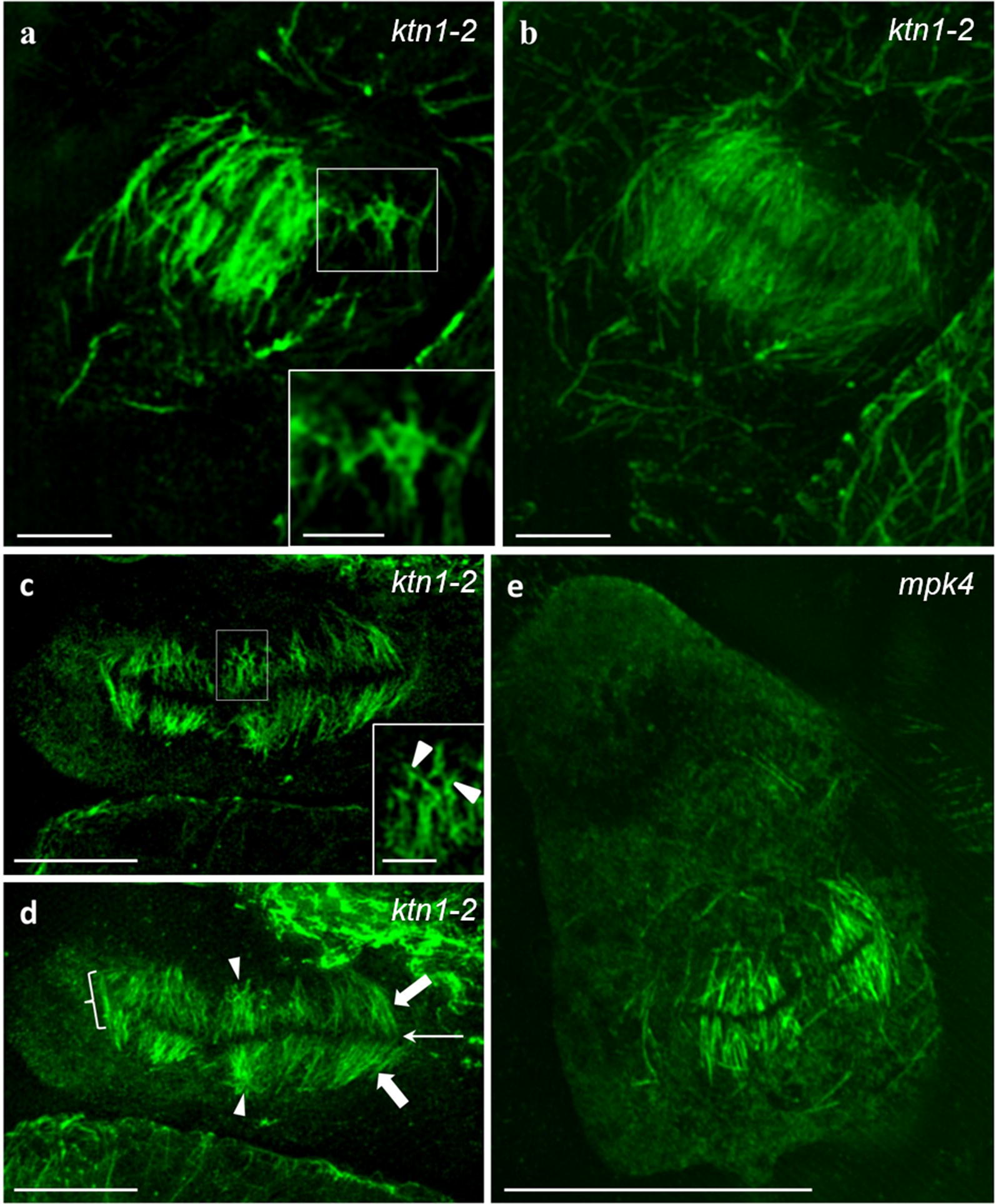
Observation of abnormal phragmoplast formation in the *ktn1*-*2* and *mpk4* mutants. **a**, **b** Single optical section (**a**) and maximum intensity projection (**b**) of an early phragmoplast of a *ktn1*-*2* cytokinetic root cell, showing an area with extensively branched microtubules (**a**, inset) at the phragmoplast periphery. **c**, **d** Single optical section (**c**) and maximum intensity projection (**d**) of an aberrant late phragmoplast of the *ktn1*-*2* mutant. Excessive microtubule branching (arrowheads) is observed within the phragmoplast (**a**, **c**; inset). **e** Ectopic and abortive phragmoplast of an aberrant root epidermal cell of the *mpk4* mutant. Bars in **e** = 10 μm; **c**, **d** = 5 μm; **a**, **b** = 2 μm; insets of **a** and **c** = 1 μm

Detailed observation of early (Fig. 4a, b) or late (Fig. 4c, d) cytokinetic cells of *ktn1*-*2* revealed the occurrence of multiple events of de novo branched formation of microtubules within the phragmoplast (Fig. 4a, c, insets) and finally we documented abortive phragmoplasts of *mpk4* mutant (e.g., Fig. 4e).

### Using SIM for colocalization studies

SIM might improve colocalization studies since its lateral resolution is at least two-fold better than that of the CLSM. In the next sections we demonstrate examples of erroneous colocalizations deciphered by CLSM and how they are corrected by SIM, while we also show how SIM can provide spatial interrelations at minute details.

### Localization of MAP65-3 with cytokinetic microtubules

MAP65-3 is a microtubule crosslinking protein whose expression is restricted to mitotic stages, whereby it is associated conspicuously with telophase and cytokinetic microtubular systems [17]. MAP65-3 is of particular interest in the study of plant cytokinesis since its absence is associated with severe cytokinetic phenotypes [30], while its function is subjected to regulation via MAPKs or phosphoinositide kinases (e.g., [5]) either by means of phosphorylation [45], or by regulation of its localization [27].

In the absence of specific antibodies against MAP65-3, we rather localized eGFP-MAP65-3 (aminoterminal fusion of MAP65-3 with enhanced green fluorescent protein) in plant cells, using instead commercial anti-GFP nanobodies conjugated to Atto 488. Tubulin was detected with a rat monoclonal antibody and Alexa Fluor 647-conjugated anti-rat IgGs. Although a red dye (such as AlexaFluor 546 or AlexaFluor 594) is expected to yield better resolution than AlexaFluor 647, the latter exhibits an outstanding brightness, photostability and pH-tolerance (https://www.thermofisher.com/cz/en/home/life-science/cell-analysis/fluorophores/alexa-fluor-647.html), allowing for high signal-to-noise ratio labeling necessary for the efficient modulation of the sample with the light pattern. Nanobody-based detection is a relatively novel detection tool with several advantages over commercial tetrameric IgGs and presents an emerging method in the plant field (e.g., [40]).

**Fig. 5:**
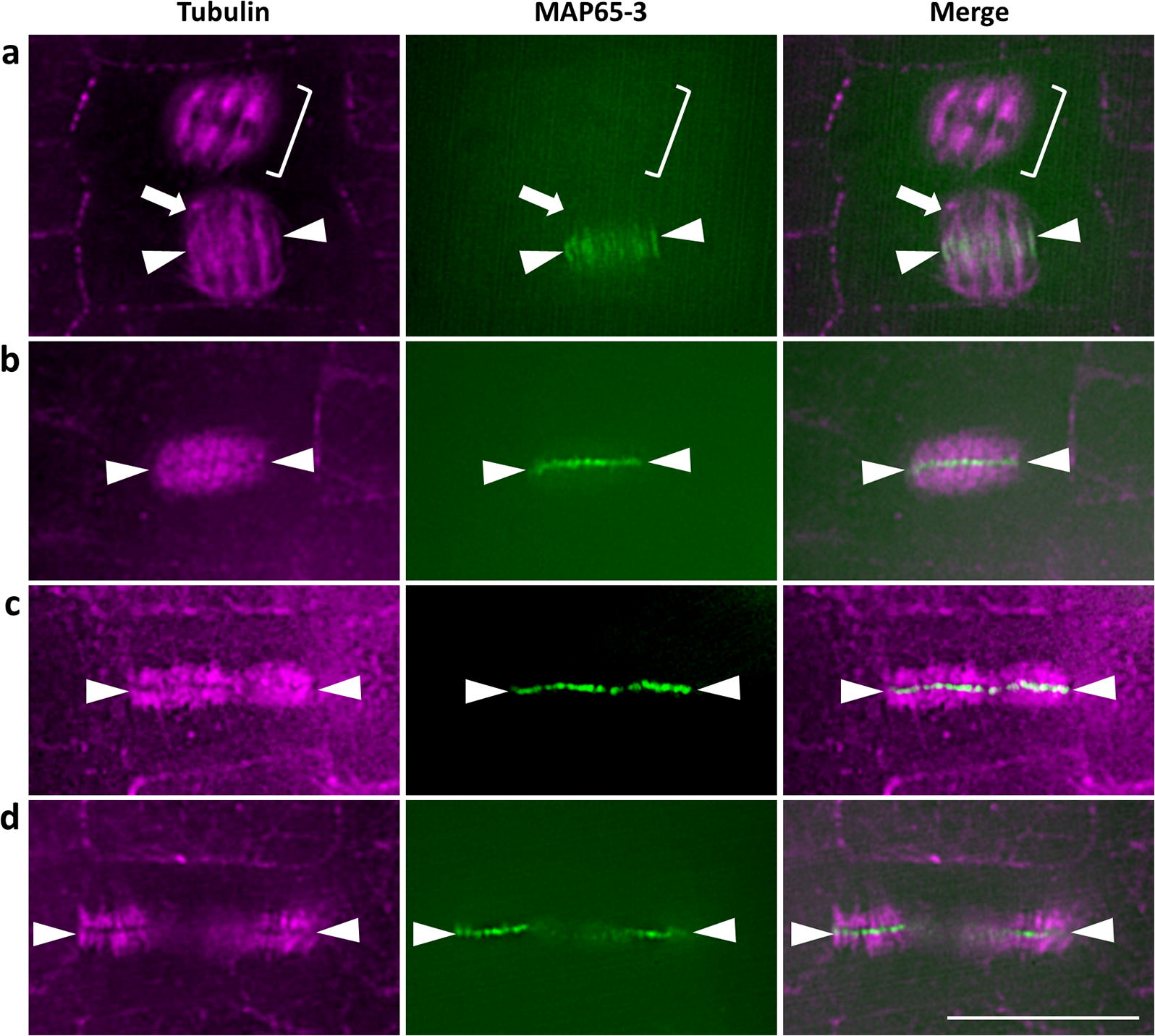
Differential localization of eGFP-MAP65-3 with mitotic and cytokinetic microtubule systems. **a** Microtubule, MAP65-3 and overlay of a metaphase spindle (top bracket) devoid of MAP65-3 signal and a telophase spindle (arrow) showing broad accumulation of MAP65-3 at the midplane (arrowheads). **b** Colocalization of microtubules and MAP65-3 at the midzone (arrowheads). **c** A more advanced phragmoplast with similar midplane distribution of MAP65-3 (arrowheads). **d** Restriction of MAP65-3 signal at phragmoplast edges (arrowheads) and its disappearance from central areas of the phragmoplast devoid of microtubules. Bars in **a**–**d** = 10 μm

MAP65-3 starts to colocalize with microtubules during telophase (Fig. 5a; bottom spindle; arrow) as at earlier stages, mitotic spindles are devoid of MAP65-3 signal (Fig. 5a; top spindle; bracket) and the telophase signal of MAP65-3 corresponds to a rather broad band around the spindle midzone (Fig. 5a; arrowheads). During cytokinesis, MAP65-3 became narrowly localized to the midplane of the phragmoplast (coinciding with the position of cell plate; Fig. 5b–d; arrowheads).

**Fig. 6:**
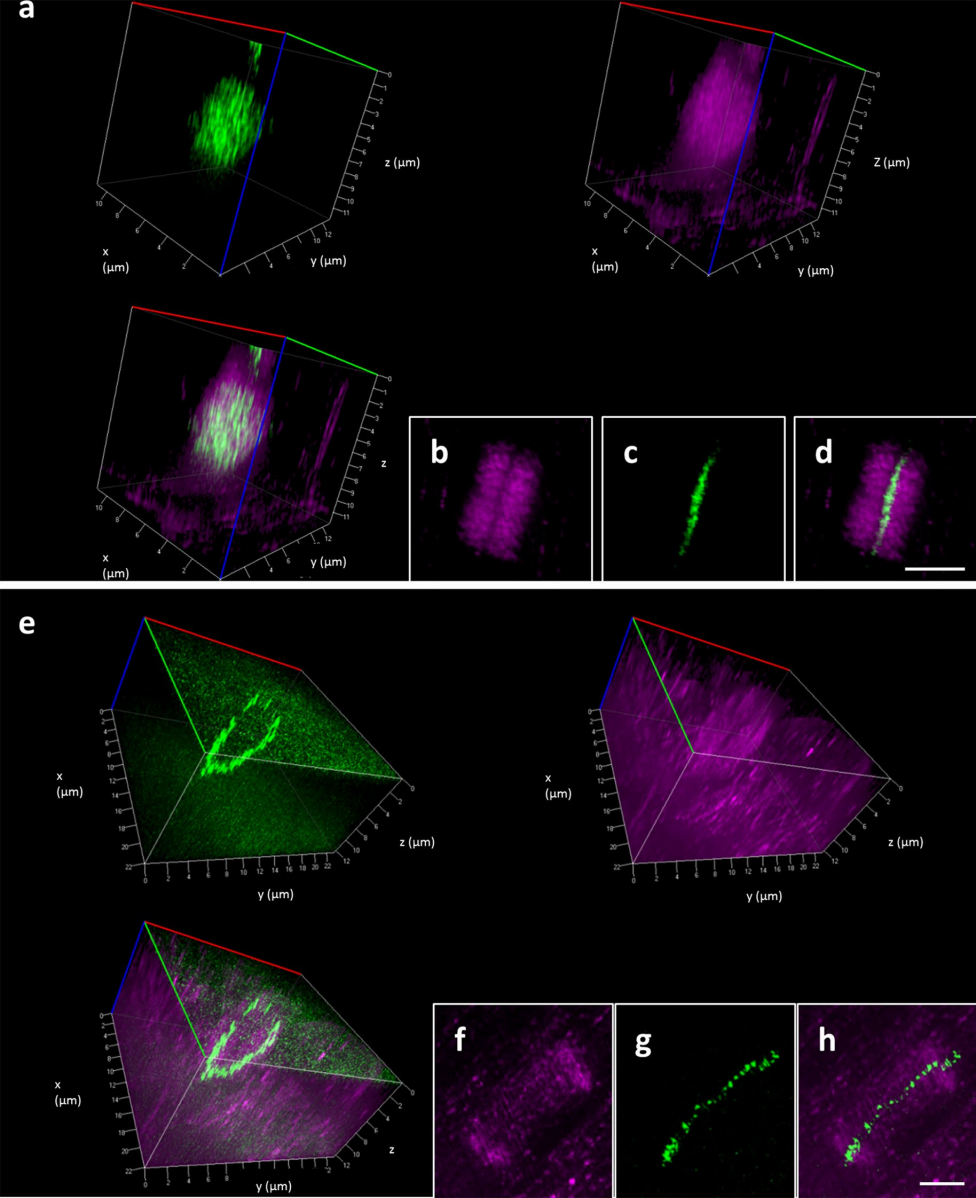
2-color 3D-SIM of MAP65-3 and microtubule distribution in cytokinesis. **a** Clockwise 3D rendering of MAP65-3, microtubules and their overlay in an early phragmoplast wherein both signals occupy the entire surface of the phragmoplast (corresponding to Additional file 4: Video 4). **b**–**d** Maximum intensity projection showing microtubule (**b**), MAP65-3 (**c**) and their overlay (**d**) in the same phragmoplast as in **a**. **e** Clockwise 3D rendering of MAP65-3, microtubules and their overlay in an late phragmoplast wherein both signals are restricted in the edges of the phragmoplast (corresponding to Additional file 5: Video 5). **f**–**h** Maximum intensity projection showing microtubule (**f**), MAP65-3 (**g**) and their overlay (**h**) in the same phragmoplast as in **e**. Bars = 2 μm

The phragmoplast is a cellular structure that expands considerably during the course of cytokinesis, therefore it was a challenge to see whether SIM could be used to visualize cytokinesis at different stages. As examples we used again cells co-immunolabeled for microtubules and eGFP-MAP65-3 and documented their distribution during early (Fig. 6a–d; Additional file 4: Video 4) and late (Fig. 6e–h; Additional file 5: Video 5) stages of phragmoplast formation. This experiment revealed that in contrast to 2D imaging of either single optical sections (Fig. 5), or maximum intensity projections (Fig. 6b–d, f–h), 3D-SIM was able to show differences in the distribution of the two signals during different stages of the phragmoplast expansion.

### Nuclear localization of MPK6 and active MAPK species

MAPKs are known to shuttle between the nucleus and the cytoplasm on an activity-based manner (reviewed in [38]). Therefore, it is reasonable to assume that a nuclear resident MAPK might be active, and this can be addressed by colocalizing the MAPK itself by a specific antibody and by an activation-pendant (phospho-specific) antibody. To test this hypothesis we colocalized MPK6, previously shown to have a predominant nuclear localization [53] and activated plant MAPKs recognized by antibody against dually phosphorylated extracellular signal related protein kinases 1 and 2 of mammalian cells (ERK1/2). The antibody against MPK6 recognizes a 14-aminoacid carboxylterminal epitope, while the anti-pERK antibody recognizes a restricted epitope which encompasses the TEY motif present in MPK6 and other MAPKs [18], only when this motif is dually phosphorylated (https://www.cellsignal.com/products/primary-antibodies/phospho-p44-42-mapk-erk1-2-thr202-tyr204-antibody/9101).

**Fig. 7:**
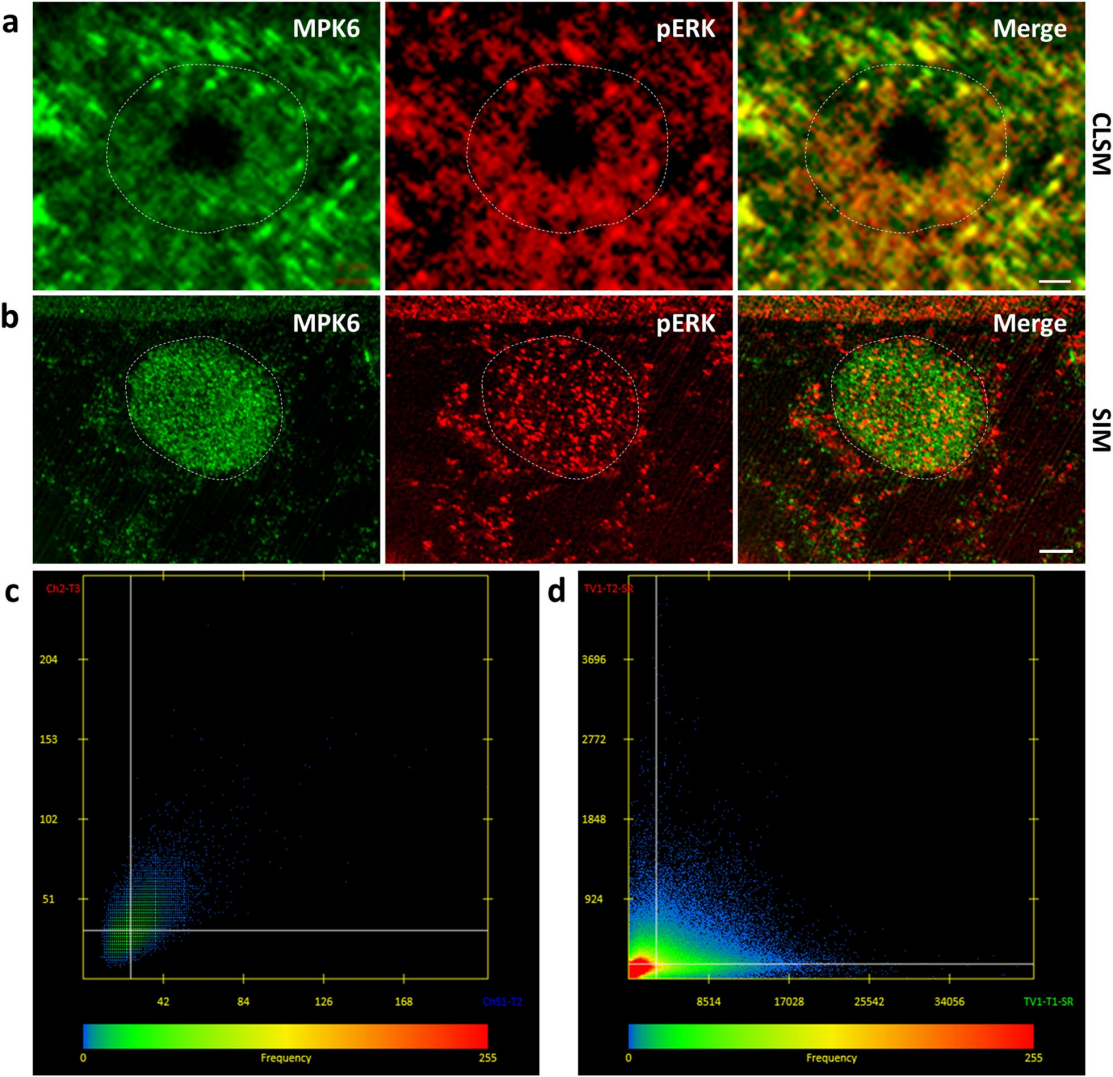
Quantitative colocalization of MPK6 and pERK by means of CLSM and SIM. **a** Nuclear colocalization of MPK6 (**a**; left panel), pERK species (**a**; middle panel) and their overlay (**a**; right panel) after CLSM documentation. **b** Nuclear colocalization of MPK6 (**b**; left panel), pERK species (**b**; middle panel) and their overlay (**b**; right panel) after SIM documentation. Dashed white lines delineate the nucleus. **c** Fluorescence intensity correlation between the signal corresponding to MPK6 and the signal corresponding to pERK from (**a**) showing positive correlation with a Manders coefficient of 0.972. **d** Fluorescence intensity correlation between the signal corresponding to MPK6 and the signal corresponding to pERK from (**b**) showing absence of correlation with a Manders coefficient of 0. Bars = 2 μm

By means of CLSM imaging, MPK6 (Fig. 7a; left panel) and pERK species (Fig. 7a; middle panel) were found in nuclei of wild-type root cells while quantitative colocalization [28] showed a good correlation between the two signals (Fig. 7a; right panel) with a correlation coefficient of 0.972 (Fig. 7c). When similar samples were documented with SIM (Fig. 7b), due to the achieved higher resolution it was found that the signals owing to MPK6 (Fig. 7b; left panel) and pERK (Fig. 7b; middle panel), are not colocalizing (Fig. 7b; right panel; d). This suggests that nuclear active MAPK species are other than MPK6 and support the previous idea that active MPK6 and its closely related *Medicago sativa* homologue SIMK are exported from the nucleus when active [35, 44]. In this particular case, Manders coefficient was 0 suggesting the absence of colocalization, while inspection of the respective scatterplot shows the coincidence of red and green pixels occurs behind the Costes threadlines (Fig. 7d).

### Association of cytoplasmic MAPKs with microtubule arrays

As previously shown, the MPK6 of *Arabidopsis thaliana* has a dominant nuclear localization [53] although a fraction may associate with the plasma membrane and cytoplasmic vesicular structures [31] as well as with microtubular PPBs, mitotic spindles and phragmoplasts [24, 53].

**Fig. 8:**
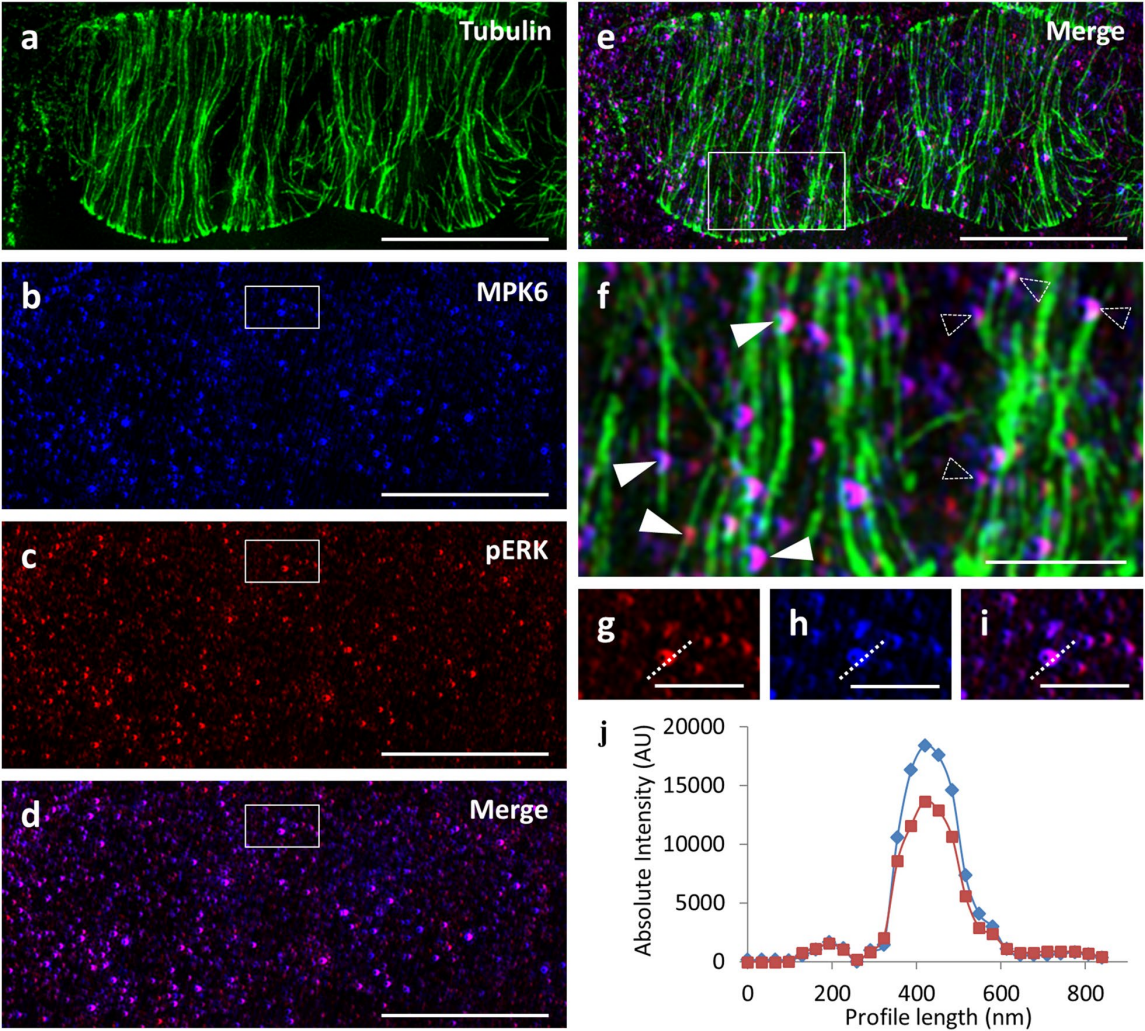
Colocalization of MPK6 and pERK in microtubule bound spots analyzed by SIM. **a**–**e** Microtubule (**a**), MPK6 (**b**), pERK (**c**), MPK6-pERK overlay (**d**) and microtubule-MPK6-pERK overlay (**e**) in root epidermal cells of Col-0. **f** Close-up view of the boxed area of **e**, showing pERK and MPK6-labeled spots associated with the walls (full arrowheads) or the tips (outlined arrowheads) of cortical microtubules. **g**–**i** MPK6 (**g**), pERK (**h**) and overlay (**i**) in spots of the outlined area of **b**–**d**. **j** Fluorescence intensity of the profile drawn in **g**–**i**, showing absolute coincidence between the signals corresponding to MPK6 and pERK. Bars in **a**–**e** = 5 μm; **c** = 2 μm; **g-i** = 1 μm

A survey of the occurrence of MPK6 in the cortical cytoplasm of interphase cells showed its conspicuous localization in vesicular structures that form intimate association with cortical microtubule walls and tips (Fig. 8a–f), while qualitative and quantitative colocalization studies showed a stunning correlation of MPK6 with the localization of pERK species (Fig. 8g–j).

## Discussion

In the present study we provide an improved version of standard immunolabeling procedures, mostly applicable to root whole-mount preparations. This protocol is designed to improve antibody penetration within the z-range limits of 3D-SIM, and preserve intracellular structure and antigenicity at the standards set by superresolution imaging. Using this protocol, it was possible to label vast intracellular structures such as the microtubule cytoskeleton, to allow for sufficient labeling density and to achieve the high SNR values required for adequate SIM. Likewise it was possible to label less abundant and more diffusely distributed structures such as MPK6 and pERK positive spots and provide a correlative output based on multichannel imaging.

The use of TDE instead of glycerol as a mountant, compensated well for refractive index mismatches and made possible the recording of Z-stacks ranging from ca. 5 µm deep, covering the axial occupancy of a typical mitotic spindle, to depths ranging from 15 to 20 µm encompassing fully expanded phragmoplasts. The amount of time necessary for such acquisitions prohibits the use of SIM to document such structures in living cells, especially in the case of multichannel documentation. The importance of mounting media in the z-range of SIM, was very recently addressed [55]. In our case TDE helped to speed up sample preparation as compared to other laborious immunofluorescence-compatible tissue clearing approaches (e.g., [37, 58]) which may additionally compromise the integrity of the structurally-labile root after enzymatic cell wall digestion. Emission intensity of fluorescent proteins is reportedly impaired in high TDE concentrations [32], and this problem was alleviated by using fluorophore-tagged anti-GFP antibodies.

We expect that other mounting media can be efficiently used for purposes of 3D SIM imaging and the ultimate choice depends on both how stable is the sample during the documentation which is an essential consideration of root whole-mount preparations which are not stably fixed but rather immersed and free-floating in a liquid mounting medium (e.g., TDE-based, glycerol- paraphenylenediamine-based or Vectashield and other mounting media commercially available).

The volumetric documentation of subcellular architecture poses several challenges related to the speed of acquisition, the possibilities of multicolor imaging and importantly the means to compensate for light scattering issues that are increased by extending the desired z-range of imaging. Superresolution methods such as 2D and 3D SIM have provided the adequate means to visualize subcellular architecture below Abbe’s limit for reasons that have been exhaustively reviewed (e.g., [23–25, 51] and references therein). However such methods require proper sample preparation procedures aiming to achieve high SNRs and sufficient tissue clearing to compensate for light scattering issues. In this sense, the choice of an appropriate mounting medium is of paramount importance. The formulation of TDE used herein has a refractive index matching that of glass and immersion oil so basically any mounting medium with this property should be used to deal with light scattering. For this reason we tried hardset Vectashield with a refractive index of 1.46 after curing (https://vectorlabs.com/amfile/file/download/file_id/1770/product_id/298/). Unfortunately, residual PBS around the roots created refractive index mismatches that compromised image quality. This was not the case with TDE which was allowed to be infiltrated at stepwise increasing concentrations until the desired one was reached.

Since fixed samples are observed, laser power and camera exposure times can be set to yield the best possible results in terms of spatial resolution. This allowed us to document mitotic and cytokinetic microtubule arrays at unprecedented quality and allowed the visualization of minute defects underlying phragmoplast formation in *ktn1*-*2* mutant. In particular, we observed multiple de novo branched microtubule formation sites and this might underlie delays previously observed during phragmoplast expansion in living cytokinetic *ktn1*-*2* cells [21]. In this respect it would be interesting to superresolve KATANIN1 localization and relate it to microtubule nucleation sites by means of SIM.

The importance of resolution in colocalization studies and especially when those are meant to be quantified was demonstrated in the case of MPK6 and pERK species in nuclei. CLSM documentation and subsequent quantification of the two signals gave the erroneous impression of tight correlation of the two signals, whereas SIM showed that the two signals are completely unrelated.

On the other hand, the sequential documentation of microtubules, MPK6 and pERK species after triple labeling revealed that spots at the cortical cytoplasm, previously identified to contain MPK6 [31], are closely associated with microtubules. Most importantly they also contain pERK species raising the possibility that this presumably vesicular fraction of MPK6 is in an active, dually phosphorylated state. It will be of interest to follow up such a study and reveal substrates of MPK6 involved in vesicular trafficking.

## Conclusions/future prospects

Plant tissues impose severe obstacles in the microscopic documentation of intracellular structures, owing to the contribution of the cell wall, the cortical cytoplasm and the vacuole to refractive index mismatches with the optical path of the microscope (reviewed in [52]). Such mismatches may hamper the visualization of voluminous subcellular structures such as the mitotic spindle and the expanding phragmoplast. Refractive index mismatches are particularly limiting in the case of superresolution methods such as SIM which are heavily influenced by spherical aberrations [2, 12]. We find herein that the use of relatively simple mounting media containing TDE, sufficiently rectifies the contribution of the sample to the optical path and allows to make use of the high z-range of SIM. In this way it is possible to document spatial interrelations between different subcellular compartments at the subdiffraction resolution limits offered by SIM.

Speed of acquisition is another, possibly the most limiting aspect for 3D SIM since each plane has to be documented and reconstructed from some 9–25 Moire patterns resulting by the rotations and the phase-shifting of the light pattern used to illuminate the sample. Such time concerns become even more limiting during multichannel imaging. However very recent commercial developments such as those mentioned earlier, have overcome temporal issues and now the market provides SIM modules with outstanding acquisition frame rates and the possibility of simultaneous multichannel imaging.

## Materials and methods

### Plant material

*Arabidopsis thaliana* seedlings were used throughout. As wild-type we selected to work with the Columbia ecotype (Col-0). On the Col-0 background, we generated stably transformed lines of *proMAP65*-*3::eGFP*-*MAP65*-*3*, to express N-terminal eGFP fusions of the MAP65-3 microtubule crosslinking protein.

Moreover, we worked on two T-DNA insertion mutants, namely *ktn1*-*2* [34] and *mpk4* [5] which have been also raised in a Col-0 background. Wild-type, transformants and mutant seeds were surface-sterilized, plated on Phytagel or gellan gum solidified half-strength Murashige–Skoog (MS) basal salt mixture supplemented with 1% w/v sucrose as a carbon source, stratified at 4 °C for 1–4 days and finally allowed to germinate at controlled environmental conditions. Seedlings between 3 and 4 days after germination were selected for subsequent whole-mount immunolocalization experiments.

### Molecular cloning and genetic crosses

The construct for N-terminal EGFP fusion of AtMAP65-3 was developed using the binary destination vector pGWB502 [33]. Initially, a multiple cloning site linker, generated by annealing of complementary oligonucleotides pGWB502link-F (ggttaattaatcctaggaatctgaagcactgccttgtggtacctgagct) and pGWB502link-R (caggtaccacaaggcagtgcttcagattcctaggattaattaacctgca), was cloned into SbfI and SacI digested pGWB502, in order to replace vector fragment containing CaMV 35 promoter and Gateway^®^ cloning cassette. The resulting vector was designated pGWB502link. Subsequently, a 1239 bp DNA fragment of MAP65-3 native promoter region was amplified from wild-type Col-0 genomic DNA using PCR with primers pMAP65-3-F (ctctcttaattaaacactcttccctacacaaaaccg) and pMAP65-3-R (aggtctaggtaccttcgaaatgcttaagcctgtaac), which contain PacI and Acc65I restriction site, respectively. Resulting PCR product was digested with PacI and Acc65I and cloned into PacI and Acc65I digested vector pGWB502link to generate construct pGWB502link-pMAP65-3. An open reading frame of MAP65-3 including stop codon was amplified from wild-type Col-0 cDNA using PCR with primers MAP65-3cDNA-F (acactagggtaccatggcaagtgttcaaaaagatcc) and MAP65-3cDNA-R (agatctacgtacgggatcctcaaaccaaacgacattcagact), which contain Acc65I and BsiWI restriction site, respectively. Obtained PCR product was digested with Acc65I and BsiWI and cloned into Acc65I digested pGWB502link-pMAP65-3 to generate construct pGWB502link-pMAP65-3::MAP65-3. Acc65I and BsiWI digest different restriction sites, but produce the same sticky ends. Therefore, in pGWB502link-pMAP65-3::MAP65-3 only unique Acc65I restriction site is present in between pMAP65-3 and MAP65-3 start codon. ORF of EGFP was PCR amplified with primers EGFP-F (tcctgtcaggtaccaagaagaaaaatggtgagcaagggcgaggagctgttca) and EGFP-R (cacagtttggtacccttagcagctgcctcttttgcggcagcctctttagcagcagcttccttgtacagctcgtccatgccgagagtga) both of which contain Acc65I restriction site, while reverse primer contains no stop codon and DNA sequence encoding rigid linker (EAAAK)3, which joins EGFP tag with AtMAP65-3 [59]. Generated PCR product was digested with Acc65I and cloned into Acc65I digested pGWB502link-pMAP65-3::MAP65-3 to create construct pMAP65-3::EGFP:MAP65-3. pMAP65-3::EGFP:MAP65-3 construct was transformed into *Agrobacterium tumefaciens* strain GV3101::pMP90 and recombinant GV3101::pMP90 clones were used for simplified floral dip transformation of *Arabidopsis thaliana* Col-0 ecotype [11]. Primary transformants of *Arabidopsis thaliana* were selected on MS media (4.3 g/L MS + vitamins, 10 g sucrose/L, 500 μl/L MES, 5.7 g/L Gellan gum, pH 5.7) supplemented with 30 mg/L hygromycine (Roche).

### Chemicals

All standard chemicals were ordered from Sigma unless stated otherwise. MS basic salt mixture without vitamins was from Duchefa. Gellan gum (a substitute for Phytagel) was from ThermoFisher (Kandel) GmbH. Electron microscopy grade fixatives were from Polysciences Inc. (16% v/v methanol-free aqueous formaldehyde and 25% v/v aqueous glutaraldehyde). Cell wall digesting enzymes were from Desert Biologicals. Primary and secondary antibodies used herein are listed in Table 1. Reagents for molecular cloning are mentioned in the respective section.

**Table 1:** Labeling reagents, commercial source and dilution guide

Labeling reagent	Company/catalogue number	Dilution (diluent)
Rat monoclonal anti-α tubulin antibody (clone YOL1/34)	BioRad/MCA78G	1:300 (3% w/v BSA in PBS pH 7.3)
Alpaca anti-GFP nanobody-Atto 488 conjugated (GFP-Booster)	Chromotek/gba488	1:500 (3% w/v BSA and 0.5% w/v BSAc in PBS pH 7.4)
Rabbit polyclonal anti-MPK6 antibody	Sigma/A7104	1:750 (3% w/v BSA in PBS pH 7. 3)
Rabbit polyclonal anti-pERK antibody	Cell Signaling/9101 s	1:400 (3% w/v BSA in PBS pH 7.3)
Goat anti-rat IgGs-Alexa Fluor 488 conjugated	ThermoFischer Scientific/A11006	1:500 (3% w/v BSA in PBS pH 7.3)
Goat anti-rat IgGs-Alexa Fluor 647 conjugated	ThermoFischer Scientific/A21247	1:500 (3% w/v BSA in PBS pH 7.3)
Goat anti-rabbit IgGs-Alexa Fluor 546 conjugated	ThermoFischer Scientific/A11010	1:1000 (3% w/v BSA and 0.5% w/v BSAc in PBS pH 7.4)
Goat anti-mouse IgGs-Alexa Fluor 555 conjugated	ThermoFischer Scientific/A21422	1:500 (3% w/v BSA 3 in PBS pH 7.3)

A list of primary and secondary antibodies used in the immunolocalizations of the present study, information on their commercial availability and their dilutions used herein

### Whole-mount immunofluorescence localization

For root whole-mount immunolocalization studies of microtubules, associated proteins and MAPKs we basically followed previously published procedures [43, 46] but with notable changes which follow. Fixatives included 1.5% v/v formaldehyde and 0.1% v/v glutaraldehyde buffered with microtubule stabilizing buffer (MTSB; 25 mM K-PIPES, pH 6.8; 2.5 mM EGTA and 2.5 mM MgSO_4_ × 7H_2_O) and supplemented with 0.01% v/v Triton X-100 (at room temperature for 1 h). For fixation of seedlings expressing fusion proteins with eGFP, glutaraldehyde was omitted and formaldehyde concentration was raised to 4% v/v at the same buffer conditions.

After fixation cell walls were digested with an enzyme cocktail prepared in MTSB, comprising of 2% w/v Cellulase Onozuka R10, 0.5% w/v Cellulase Onozuka RS, 1% w/v Macerozyme R10, 1% w/v Meicelase and 0.1% w/v Pectolyase Y23 (30 min, room temperature). Subsequently seedlings were washed two times with MTSB and 2 times with PBS pH 7.4 (10 min each, room temperature) and then residual aldehyde groups were reduced by 15 min incubation in 0.1% w/v sodium borohydride (NaBH_4_; this step was omitted when glutaraldehyde was not included in the fixative). Then, samples were sequentially permeabilized with 10% v/v DMSO, 2% v/v Nonidet P40 and 0.01% v/v Triton X-100 in PBS pH 7.4 for 15 min, washed in PBS pH 7.4 and blocked overnight in 3% w/v bovine serum albumin (BSA) and 0.5% w/v polyacetylated BSA (BSAc; Aurion) in PBS pH 7.4, prior to antibody incubation.

Subsequently samples were incubated in primary antibody (Table 1) diluted in 3% w/v BSA and 0.5% w/v BSAc in PBS pH 7.4 at room temperature overnight. During the following day, samples were extensively washed in PBS pH 7.4 (6 times, 10 min each), blocked with 3% w/v BSA and 0.5% w/v BSAc in PBS pH 7.4 for 1 h and incubated with secondary antibody diluted in 3% w/v BSA and 0.5% w/v BSAc in PBS pH 7.4 overnight at 37 °C. Finally, samples were washed in plain PBS pH 7.4 (at least 6 times 10 min each), counterstained with (4′,6-diamidine-2′-phenylindole dihydrochloride) DAPI and mounted in a mounting media supplemented with 0.1% w/v para-phenylene diamine as an antifade agent. For standard imaging, mounting medium was made in 90% v/v glycerol buffered with 100 mM tris(hydroxymethyl)aminomethane-HCl pH 8.8.

For improving the z-range necessary for 3D imaging, we replaced glycerol with 2,2′-thiodiethanol (TDE) at a concentration that was shown to have matching refractive index (RI) with that of the glass coverslip and the immersion oil (97% v/v; RI = 1.52; [54]). In order to achieve adequate infiltration of TDE in root whole-mount preparations, those were incubated to an increasing gradient of TDE in 100 mM Tris–Cl pH 8.8. Briefly, samples were infiltrated sequentially with 5% v/v, 10% v/v, 20% v/v, 30% v/v, 40% v/v, 50% v/v, 75% v/v and 97% v/v TDE in 100 mM Tris–Cl pH 8.8 for 10 min at room temperature per step. At the final step the samples were infiltrated in 97% v/v TDE in 100 mM Tris–Cl pH 8.8 supplemented with 0.01% w/v para-phenylene diamine and were prepared for microscopy.

We also tested hard set Vectashield (ThermoFischer Scientific) as a potential mounting medium but in our hands it introduced considerable spherical aberrations, light scattering and substandard image quality at acquisition.

### Microscopy, image acquisition and processing

Microscopy of all samples described was done using an Elyra PS.1 (Zeiss) platform using settings as described in previously published works [22, 25]. Samples were mostly documented using a 63×/1.40 NA (numerical aperture) oil immersion objective and rarely a 100×/1.46 NA oil immersion objective. AlexaFluor 488, Atto 488, eGFP were excited with a 488 nm laser line. AlexaFluor 546 and AlexaFluor 555 were excited with a 561 nm laser line and AlexaFluor 647 and Atto 647 N were excited with a 642 nm laser line. For imaging of AlexaFluor 488 and Atto 488 a band pass emission filter (495–550 nm). For AlexaFluor 546 and AlexaFluor 555 we used a band pass emission filter (570–620 nm). Finally for AlexaFluor 647 and Atto 647 N we used a long pass filter (655 nm). Especially for 3D imaging, the grating pattern was rotated 5 times (at 72° increments) with 5 additional phase shifts (at 3π/2 increments) per angular position. In all cases, laser power and camera exposure time was adjusted appropriately to ensure high SNR, and visible modulation of the sample with the emission light pattern (in this case this is achieved when the grating is visible on the screen during the acquisition).

For z-sampling the Nyquist criterion was applied for the channel with the smallest λexc (in this particular case the channel corresponding to AlexaFluor 488 and Atto 488). Since the sampling rate of this channel is higher than that required for red (AlexaFluor 546) or far-red (AlexaFluor 647) the respective channels were oversampled. All images were acquired with a PCO.Edge 5.5 scientific complementary metal-oxide semiconductor (sCMOS) camera with exposure times ranging between 80 and 500 ms.

Raw images were reconstructed using the appropriate tool of Zeiss Zen software (Black version with Structured Illumination module) using settings as previously described [25]. Especially Wiener filtering was adjusted appropriately to avoid commonly occurring artefacts such as ringing and honeycomb background [12, 25]. As a rule of thumb, relatively high filter values (ranging from − 7.0 to − 5.2) were chosen for 2D snaps, whereas lower filter values (ranging from − 5.0 to − 4.2) were chosen for entire Z-stacks. Based on full width at half maximum values of transverse fluorescence intensity profiles drawn along individual microtubules (as in [22]), XY-resolution was never worse than ca. 150 nm.

To demonstrate the angular distribution of cortical microtubules in example images of Col-0, *mpk4* and *ktn1*-*2*, we used the stand-alone software Cytospectre ([20]; http://www.tut.fi/cytospectre/).

In order to ensure the validity of quantitative colocalization studies, channels were aligned using the channel alignment plugin of the Zen software with TetraSpeck™ Microspheres, 0.1 µm, fluorescent blue/green/orange/dark red as a sample (Thermofischer Scientific). Alternatively the align RGB planes Image J plugin was used (https://imagej.net/Align_RGB_planes) either with TetraSpeck™ Microspheres, or with documented root whole-mount sample images. Quantitative colocalizations were done using the colocalization tool of Zeiss Zen Black or Zen 2 (Blue Version). Scatterplots were automatically thresholded accordingly [10] and for quantitative purposes the Manders coefficient was also automatically extrapolated [28].

3D rendering of reconstructed z-acquisitions was done using the appropriate tool of ImageJ, or the volume rendering plugin of Zen software. The output was saved as an *.avi file.

## Additional files

**Additional file 1: Video 1.** 360° Rotational view of the 3D rendering shown in Fig. 3i.

**Additional file 1: Video 2.** 360° Rotational view of the 3D rendering shown in Fig. 3j.

**Additional file 3: Video 3.** 360° Rotational view of the 3D rendering shown in Fig. 3l.

**Additional file 4: Video 4.** 360° Rotational view of the 3D rendering shown in Fig. 6a.

**Additional file 5: Video 5.** 360° Rotational view of the 3D rendering shown in Fig. 6e.

## Abbreviations

(d)STORM: (direct) stochastic optical reconstruction microscopy
2D/3D: two/three dimensional
BSA: bovine serum albumin
BSAc: polyacetylated bovine serum albumin
CLSM: confocal laser scanning microscopy
DAPI: 4′,6-diamidine-2′-phenylindole dihydrochloride
DMSO: dimethyl sulfoxide
EGTA: ethylene glycol-bis(2-aminoethylether)-*N*,*N*,*N*′,*N*′-tetraacetic acid
ERK: extracellular signal related protein kinase
FOV: field of view
(e)GFP: (enhanced) green fluorescent protein
MAP65: microtubule associated protein of 65 kDa molecular weight
MAPK: mitogen activated protein kinase
MS: Murashige–Skoog
MTSB: microtubule stabilizing buffer
NA: numerical aperture
PALM: photoactivation localization microscopy
PBS: phosphate buffered saline
PIPES: 1,4-piperazinediethanesulfonic acid
sCMOS: scientific complementary metal-oxide semiconductor
SIM: structured illumination microscopy
SNR: signal to noise ratio
STED: stimulated emission depletion
TDE: 2,2′-thiodiethanethiol
TIRF: total internal reflection

## References

[1] Antosch M, Schubert V, Holzinger P, Houben A, Grasser KD (2015). Mitotic lifecycle of chromosomal 3xHMG-box proteins and the role of their N-terminal domain in the association with rDNA loci and proteolysis. New Phytol.

[2] Arigovindan M, Sedat JW, Agard DA (2012). Effect of depth dependent spherical aberrations in 3D structured illumination microscopy. Opt Express.

[3] Banaei-Moghaddam AM, Schubert V, Kumke K, Weiß O, Klemme S, Nagaki K, Macas J, González-Sánchez M, Heredia V, Gómez-Revilla D, González-García M, Vega JM, Puertas MJ, Houben A (2012). Nondisjunction in favor of a chromosome: the mechanism of rye B chromosome drive during pollen mitosis. Plant Cell.

[4] Baroux C, Schubert V, Baroux MBC (2018). Technical review: microscopy and image processing tools to analyze plant chromatin: practical considerations. Plant chromatin dynamics: methods and protocols.

[5] Beck M, Komis G, Müller J, Menzel D, Samaj J (2010). Arabidopsis homologs of nucleus- and phragmoplast-localized kinase 2 and 3 and mitogen-activated protein kinase 4 are essential for microtubule organization. Plant Cell.

[6] Beck M, Komis G, Ziemann A, Menzel D, Samaj J (2011). Mitogen-activated protein kinase 4 is involved in the regulation of mitotic and cytokinetic microtubule transitions in *Arabidopsis thaliana*. New Phytol.

[7] Betzig E, Patterson GH, Sougrat R, Lindwasser OW, Olenych S, Bonifacino JS, Davidson MW, Lippincott-Schwartz J, Hess HF (2006). Imaging intracellular fluorescent proteins at nanometer resolution. Science.

[8] Cole RW, Jinadasa T, Brown CM (2011). Measuring and interpreting point spread functions to determine confocal microscope resolution and ensure quality control. Nat Protoc.

[9] Combs CA (2010). Fluorescence microscopy: a concise guide to current imaging methods. Curr Protoc Neurosci.

[10] Costes SV, Daelemans D, Cho EH, Dobbin Z, Pavlakis G, Lockett S (2004). Automatic and quantitative measurement of protein-protein colocalization in live cells. Biophys J.

[11] Davis AM, Hall A, Millar AJ, Darrah C, Davis SJ (2009). Protocol: streamlined sub-protocols for floral-dip transformation and selection of transformants in *Arabidopsis thaliana*. Plant Methods.

[12] Demmerle J, Innocent C, North AJ, Ball G, Müller M, Miron E, Matsuda A, Dobbie IM, Markaki Y, Schermelleh L (2017). Strategic and practical guidelines for successful structured illumination microscopy. Nat Protoc.

[13] Fišerová J, Efenberková M, Sieger T, Maninová M, Uhlířová J, Hozák P (2017). Chromatin organization at the nuclear periphery as revealed by image analysis of structured illumination microscopy data. J Cell Sci.

[14] Fitzgibbon J, Bell K, King E, Oparka K (2010). Super-resolution imaging of plasmodesmata using three-dimensional structured illumination microscopy. Plant Physiol.

[15] Gustafsson MG (2000). Surpassing the lateral resolution limit by a factor of two using structured illumination microscopy. J Microsc.

[16] Hell SW, Wichmann J (1994). Breaking the diffraction resolution limit by stimulated emission: stimulated-emission-depletion fluorescence microscopy. Opt Lett.

[17] Ho CM, Hotta T, Guo F, Roberson RW, Lee YR, Liu B (2011). Interaction of antiparallel microtubules in the phragmoplast is mediated by the microtubule-associated protein MAP65-3 in Arabidopsis. Plant Cell.

[18] Jonak C, Okrész L, Bögre L, Hirt H (2002). Complexity, cross talk and integration of plant MAP kinase signalling. Curr Opin Plant Biol.

[19] Jonkman J, Brown CM (2015). Any way you slice it—a comparison of confocal microscopy techniques. J Biomol Tech.

[20] Kartasalo K, Pölönen RP, Ojala M, Rasku J, Lekkala J, Aalto-Setälä K, Kallio P (2015). CytoSpectre: a tool for spectral analysis of oriented structures on cellular and subcellular levels. BMC Bioinform.

[21] Komis G, Luptovčiak I, Ovečka M, Samakovli D, Šamajová O, Šamaj J (2017). Katanin effects on dynamics of cortical microtubules and mitotic arrays in *Arabidopsis thaliana* revealed by advanced live-cell imaging. Front Plant Sci.

[22] Komis G, Mistrik M, Samajová O, Doskočilová A, Ovečka M, Illés P, Bartek J, Samaj J (2014). Dynamics and organization of cortical microtubules as revealed by superresolution structured illumination microscopy. Plant Physiol.

[23] Komis G, Mistrik M, Šamajová O, Ovečka M, Bartek J, Šamaj J (2015). Superresolution live imaging of plant cells using structured illumination microscopy. Nat Protoc.

[24] Komis G, Novák D, Ovečka M, Šamajová O, Šamaj J (2018). Advances in imaging plant cell dynamics. Plant Physiol.

[25] Komis G, Šamajová O, Ovečka M, Šamaj J (2015). Super-resolution microscopy in plant cell imaging. Trends Plant Sci.

[26] Kraus F, Miron E, Demmerle J, Chitiashvili T, Budco A, Alle Q, Matsuda A, Leonhardt H, Schermelleh L, Markaki Y (2017). Quantitative 3D structured illumination microscopy of nuclear structures. Nat Protoc.

[27] Lin F, Krishnamoorthy P, Schubert V, Hause G, Heilmann M, Heilmann I (2019). A dual role for cell plate-associated PI4Kbeta in endocytosis and phragmoplast dynamics during plant somatic cytokinesis. EMBO J.

[28] Manders EM, Stap J, Brakenhoff GJ, van Driel R, Aten JA (1992). Dynamics of three-dimensional replication patterns during the S-phase, analysed by double labelling of DNA and confocal microscopy. J Cell Sci.

[29] Marques A, Schubert V, Houben A, Pedrosa-Harand A (2016). Restructuring of holocentric centromeres during meiosis in the plant *Rhynchospora pubera*. Genetics.

[30] Müller S, Fuchs E, Ovecka M, Wysocka-Diller J, Benfey PN, Hauser MT (2002). Two new loci, PLEIADE and HYADE, implicate organ-specific regulation of cytokinesis in Arabidopsis. Plant Physiol.

[31] Müller J, Beck M, Mettbach U, Komis G, Hause G, Menzel D, Samaj J (2010). Arabidopsis MPK6 is involved in cell division plane control during early root development, and localizes to the pre-prophase band, phragmoplast, trans-Golgi network and plasma membrane. Plant J.

[32] Musielak TJ, Slane D, Liebig C, Bayer M (2016). A versatile optical clearing protocol for deep tissue imaging of fluorescent proteins in *Arabidopsis thaliana*. PLoS ONE.

[33] Nakagawa T, Suzuki T, Murata S, Nakamura S, Hino T, Maeo K (2007). Improved gateway binary vectors: high-performance vectors for creation of fusion constructs in transgenic analysis of plants. Biosci Biotechnol Biochem.

[34] Nakamura M, Ehrhardt DW, Hashimoto T (2010). Microtubule and katanin-dependent dynamics of microtubule nucleation complexes in the acentrosomal Arabidopsis cortical array. Nat Cell Biol.

[35] Ovečka M, Takáč T, Komis G, Vadovič P, Bekešová S, Doskočilová A, Šamajová V, Luptovčiak I, Samajová O, Schweighofer A, Meskiene I, Jonak C, Křenek P, Lichtscheidl I, Škultéty L, Hirt H, Šamaj J (2014). Salt-induced subcellular kinase relocation and seedling susceptibility caused by overexpression of Medicago SIMKK in Arabidopsis. J Exp Bot.

[36] Ovečka M, von Wangenheim D, Tomančák P, Šamajová O, Komis G, Šamaj J (2018). Multiscale imaging of plant development by light-sheet fluorescence microscopy. Nat Plants.

[37] Palmer WM, Martin AP, Flynn JR, Reed SL, White RG, Furbank RT, Grof CP (2015). PEA-CLARITY: 3D molecular imaging of whole plant organs. Sci Rep.

[38] Plotnikov A, Zehorai E, Procaccia S, Seger R (2011). The MAPK cascades: signaling components, nuclear roles and mechanisms of nuclear translocation. Biochim Biophys Acta.

[39] Ribeiro T, Marques A, Novák P, Schubert V, Vanzela AL, Macas J, Houben A, Pedrosa-Harand A (2017). Centromeric and non-centromeric satellite DNA organisation differs in holocentric Rhynchospora species. Chromosoma.

[40] Rocchetti A, Hawes C, Kriechbaumer V (2014). Fluorescent labelling of the actin cytoskeleton in plants using a cameloid antibody. Plant Methods.

[41] Rust MJ, Bates M, Zhuang X (2006). Sub-diffraction-limit imaging by stochastic optical reconstruction microscopy (STORM). Nat Methods.

[42] Sahl SJ, Hell SW, Jakobs S (2017). Fluorescence nanoscopy in cell biology. Nat Rev Mol Cell Biol.

[43] Samajová O, Komis G, Samaj J (2014). Immunofluorescent localization of MAPKs and colocalization with microtubules in Arabidopsis seedling whole-mount probes. Methods Mol Biol.

[44] Šamaj J, Ovečka M, Hlavacka A, Lecourieux F, Meskiene I, Lichtscheidl I, Lenart P, Salaj J, Volkmann D, Bögre L, Baluska F, Hirt H (2002). Involvement of the mitogen-activated protein kinase SIMK in regulation of root hair tip growth. EMBO J.

[45] Sasabe M, Kosetsu K, Hidaka M, Murase A, Machida Y (2011). *Arabidopsis thaliana* MAP65-1 and MAP65-2 function redundantly with MAP65-3/PLEIADE in cytokinesis downstream of MPK4. Plant Signal Behav.

[46] Sauer M, Paciorek T, Benková E, Friml J (2006). Immunocytochemical techniques for whole-mount in situ protein localization in plants. Nat Protoc..

[47] Schmid VJ, Cremer M, Cremer T (2017). Quantitative analyses of the 3D nuclear landscape recorded with super-resolved fluorescence microscopy. Methods.

[48] Schubert V, Lermontova I, Schubert I (2014). Loading of the centromeric histone H3 variant during meiosis—how does it differ from mitosis?. Chromosoma.

[49] Schubert V, Lermontova I, Schubert I (2013). The Arabidopsis CAP-D proteins are required for correct chromatin organisation, growth and fertility. Chromosoma.

[50] Schubert V (2014). RNA polymerase II forms transcription networks in rye and Arabidopsis nuclei and its amount increases with endopolyploidy. Cytogenet Genome Res.

[51] Schubert V (2017). Super-resolution microscopy—applications in plant cell research. Front Plant Sci.

[52] Shaw SL, Ehrhardt DW (2013). Smaller, faster, brighter: advances in optical imaging of living plant cells. Annu Rev Plant Biol.

[53] Smékalová V, Luptovčiak I, Komis G, Šamajová O, Ovečka M, Doskočilová A, Takáč T, Vadovič P, Novák O, Pechan T, Ziemann A, Košútová P, Šamaj J (2014). Involvement of YODA and mitogen activated protein kinase 6 in Arabidopsis post-embryogenic root development through auxin up-regulation and cell division plane orientation. New Phytol.

[54] Staudt T, Lang MC, Medda R, Engelhardt J, Hell SW (2007). 2,2′-thiodiethanol: a new water soluble mounting medium for high resolution optical microscopy. Microsc Res Tech.

[55] Szczurek A, Contu F, Hoang A, Dobrucki J, Mai S (2018). Aqueous mounting media increasing tissue translucence improve image quality in structured illumination microscopy of thick biological specimen. Sci Rep.

[56] Török P, Hewlett SJ, Varga P (1997). The role of specimen-induced spherical aberration in confocal microscopy. J Microsc.

[57] Vyplelová P, Ovečka M, Komis G, Šamaj J (2018). Advanced microscopy methods for bioimaging of mitotic microtubules in plants. Methods Cell Biol.

[58] Warner CA, Biedrzycki ML, Jacobs SS, Wisser RJ, Caplan JL, Sherrier DJ (2014). An optical clearing technique for plant tissues allowing deep imaging and compatible with fluorescence microscopy. Plant Physiol.

[59] Werner S, Marillonnet S, Hause G, Klimyuk V, Gleba Y (2006). Immunoabsorbent nanoparticles based on a tobamovirus displaying protein A. Proc Natl Acad Sci USA.

